# Hypoxia-inducible miR-182 enhances HIF1α signaling via targeting PHD2 and FIH1 in prostate cancer

**DOI:** 10.1038/srep12495

**Published:** 2015-07-24

**Authors:** Yan Li, Duo Zhang, Xiaoyun Wang, Xuan Yao, Cheng Ye, Shengjie Zhang, Hui Wang, Cunjie Chang, Hongfeng Xia, Yu-cheng Wang, Jing Fang, Jun Yan, Hao Ying

**Affiliations:** 1 Key Laboratory of Food Safety Research, Institute for Nutritional Sciences, Shanghai Institutes for Biological Sciences, University of Chinese Academy of Sciences, Shanghai 200031, China; 2 Model Animal Research Center, and MOE Key Laboratory of Model Animals for Disease Study, Nanjing University, Nanjing 210061, China; 3 Department of Nutrition, Shanghai Xuhui Central Hospital, Shanghai 200031, China; 4 Clinical Research Center of Institute for Nutritional Sciences, Shanghai Institutes for Biological Sciences, Chinese Academy of Sciences, Shanghai 200031, China; 5 Key Laboratory of Food Safety Risk Assessment, Ministry of Health, Beijing, China

## Abstract

Activation of hypoxia-inducible factor 1α (HIF1α) controls the transcription of genes governing angiogenesis under hypoxic condition during tumorigenesis. Here we show that hypoxia-responsive miR-182 is regulated by HIF1α at transcriptional level. Prolyl hydroxylase domain enzymes (PHD) and factor inhibiting HIF-1 (FIH1), negative regulators of HIF1 signaling, are direct targets of miR-182. Overexpression of miR-182 in prostate cancer cells led to a reduction of PHD2 and FIH1 expression and an increase in HIF1α level either under normoxic or hypoxic condition. Consistently, inhibition of miR-182 could increase PHD2 and FIH1 levels, thereby reducing the hypoxia-induced HIF1α expression. Matrigel plug assay showed that angiogenesis was increased by miR-182 overexpression, and vice versa. miR-182 overexpression in PC-3 prostate cancer xenografts decreased PHD2 and FIH1 expression, elevated HIF1α protein levels, and increased tumor size. Lastly, we revealed that the levels of both miR-182 and HIF1α were elevated, while the expression PHD2 and FIH1 was downregulated in a mouse model of prostate cancer. Together, our results suggest that the interplay between miR-182 and HIF1α could result in a sustained activation of HIF1α pathway, which might facilitate tumor cell adaption to hypoxic stress during prostate tumor progression.

Prostate cancer has become one of the most common cancers in men worldwide. According to the estimation from the American Cancer Society, about 1 in 7 males will be diagnosed with prostate cancer during the lifetime. Despite substantial progress in therapies, prostate cancer is still the second leading cause of cancer-related death in American men. Although factors of both genes and environment are common causes of prostate cancer development and progression, the underlying molecular mechanisms are not very clear. Therefore, better understanding the pathogenesis of prostate cancer and exploring novel intervention targets are urgent.

Hypoxia is a hallmark of cancer^1^. Cancer cells have the ability to adapt to hypoxic environments by changing cellular metabolism and increasing vascularization^2^,^3^. Hypoxia is also a common feature of prostate tumors associated with poor prognosis^4^. Increasing levels of hypoxia correlate significantly with increasing clinical stage^5^. Tumor aggressiveness and poor patient survival are associated with microvessel density in prostate cancer^6^,^7^. Due to the role of hypoxia in prostate cancer progression, the key players in hypoxic adaptation and angiogenesis have been considered as drug targets for prostate cancer prevention and management^8^.

One of the master regulators of cellular response to hypoxia is hypoxia-inducible factor 1 (HIF1), a heterodimeric transcription factor composed of a hypoxia-inducible HIF1α and a stably expressed HIF1β^9^. HIF1α protein stability is controlled by the oxygen sensing prolyl hydroxylase domain (PHD) enzymes while its transcriptional activity is regulated by the asparaginyl hydroxylase FIH (factor inhibiting HIF-1)^10^,^11^. In addition, PHD2, the main celluar oxygen level sensor, is a direct HIF target gene in human hepatoma cells, ovarial carcinoma cells and osteosarcoma cells^12^. Growing evidence suggests that HIF1α stability and/or activity could be modulated by oncogene activation, loss of tumor suppressors, and metabolites such as succinate, fumarate, and free radicals^13^,^14^.

It has been shown that HIF1α is involved in hypoxic adaptation and angiogenesis during cancer progression^15^. HIF-1α is overexpressed in primary and metastatic prostate cancers^16^. The upregulation of HIF-1α is an early event in prostate carcinogenesis and is associated with the angiogenic switch^17^. More importantly, overexpression of HIF1α increases the risk of castration resistance and metastases in prostate cancer^18^. Currently, inhibitors for HIF1α have been developed for clinical use.

miRNAs constitute a novel class of non-coding RNAs which fine-tune the gene expression at the post-transcriptional level^19^. Emerging evidences suggest that miRNAs are also involved in HIF1α-mediated hypoxia adaption and angiogenesis. For example, miR-210 is hypoxia-inducible and could affect HIF1α stability in various cell lines^20^,^21^,^22^. miR-31 activates HIF1 pathway in head and neck carcinoma by inhibiting FIH^23^. In endothelial cells, miR-424 could stabilize HIFα and promote angiogenesis, while Let-7 and miR-103/107 could enhance angiogenesis by targeting argonaute 1 (AGO1)^24^,^25^. However, whether miRNAs could directly target the key components of HIFα pathway and affect prostate tumor angiogenesis is not fully elucidated. Understanding the miRNA regulatory network in HIF1α-controlled hypoxia response will provide not only new insight into hypoxia adaption and angiogenesis but also therapeutic targets for prostate cancer.

It has been reported recently that miR-183-96-182 cluster is overexpressed in prostate cancer and is able to modulate zinc homeostasis through zinc transporter hZIP1^26^,^27^. It has been demonstrated that miR-182 overexpression promotes prostate cancer cell proliferation and invasion by targeting multiple genes, including FOXF2 (forkhead box F2), RECK (reversion-inducing-cysteine-rich protein with kazal motifs), MTSS1 (metastasis suppressor 1), and NDRG1 (N-myc downstream regulated 1)^28^,^29^. In addition, miR-182 could induce mesenchymal to epithelial transition and growth factor independent growth via repressing snail family zinc finger 2 (SNAI2) in prostate cells^30^. However, whether miR-182 is involved in hypoxia adaption or angiogenesis and the mechanism of transcriptional regulation of miR-183-96-182 are unknown.

In this study, we showed that miR-183-96-182 is hypoxia-responsive and is directly regulated by HIF1α at transcriptional level. Interestingly, we found miR-182 could enhance the expression levels of HIF1α and its target gene, vascular endothelial growth factor (VEGF), by targeting PHD2 and FIH1. We also found that angiogenesis was increased by miR-182 overexpression and suppressed by miR-182 inhibitor using the matrigel plug assay. Furthermore, we observed that overexpressing miR-182 could increase HIF1α expression and promote tumor angiogenesis in a prostate cancer xenograph model. Lastly, we showed that both miR-183-96-182 and HIF1α expression levels were upregulated in a mouse model containing a prostate-specific phosphatase and tensin homolog (PTEN) deletion (PTEN^PC−/−^ mice). Together, we provided evidence for a novel mechanism regulating HIF-1α during hypoxia adaption and angiogenesis in prostate cancer.

## Results

### miR-183-96-182 cluster is hypoxia-responsive and regulated by HIF1α

**Figure 1:**
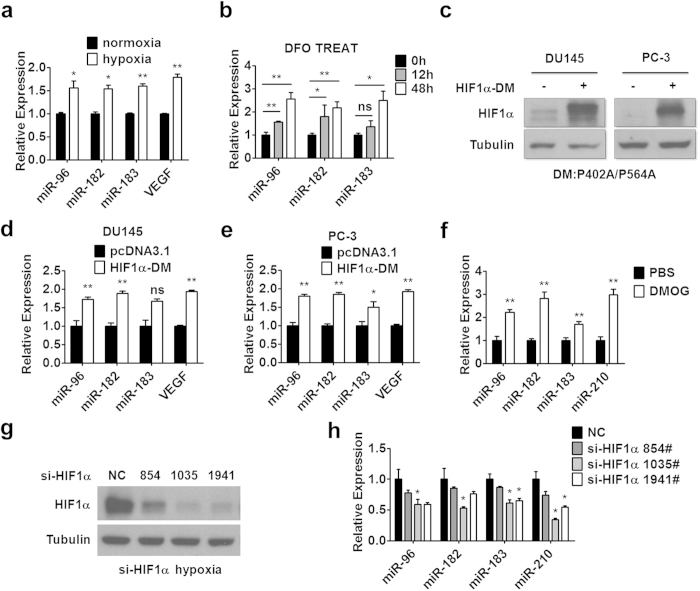
miR-183-96-182 cluster is hypoxia-responsive and regulated by HIF1α. (**a**,**b**) The expression of miR-183-96-182 was assayed by RT-PCR in PC-3 cells after exposure to 1% O_2_ hypoxic environment for 24 hours (**a**) or treated with hypoxia inducer DFO for 12 or 48 hours (**b**). (**c**) DU145 and PC-3 cells were transfected with HIF1α-DM or control empty plasmid. After 48 hours, the cells were harvested and HIF1α protein was determined. Tubulin was used as a loading control. (**d**,**e**) Relative expression of miR-183-96-182 was determined in DU145 (**d**) or PC-3 (**e**) cells after transfected with HIF1α-DM as indicated. VEGF mRNA was determined as a positive control. (**f**) C57BL/6 mice were treated with DMOG or vehicle as described in Materials and Methods. The prostate tissues were harvested and the miRNA expression was determined by RT-PCR analysis (n = 3). (**g**) PC-3 cells were transfected with control or HIF1α siRNA oligos. After 24 hours the cells were exposed to 1% O_2_ for 24 hours, then the cells were harvested and HIF1α protein was determined. (**h**) PC-3 cells were transfected with HIF1α siRNA oligos and exposed to hypoxia. The expression of miR-183-96-182 was determined by RT-PCR. miR-210 expression was also determined. Data are mean ± SEM of three independent experiments. *p < 0.05, **p < 0.01. Full-length blots are presented in Supplementary Fig. S6.

To test whether miRNAs are involved in hypoxia adaption or angiogenesis in prostate cancer, we examined the expression of a group of miRNAs in prostate cancer cell lines under hypoxic environment as described in Materials and Methods. We found that the expression levels of miR-183-96-182 as well as VEGF, a known hypoxia responsive gene, were upregulated in PC-3 prostate cancer cells under the 1% O_2_ culture condition (Fig. 1a) or after hypoxia-mimetic agent deferoxamine (DFO) treatment (Fig. 1b). DFO is a free radical scavenger and iron chelator, DFO could stop HIF1α hydroxylation, thereby inducing HIF1α accumulation even under the condition of normoxia^31^. Since HIF1α is a master transcriptional regulator in response to hypoxia, we determined whether HIF1α could regulate the transcription of miR-183-96-182 by using a HIF1α double mutant (HIF1α-DM) that could not be hydroxylated by PHDs for degradation. As shown in Fig. 1c, transfection of HIF1α-DM resulted in an accumulation of HIF1α in DU145 and PC-3 prostate cancer cells under normoxia. Meanwhile, the expression of miR-183-96-182 and HIF1α target gene VEGF, was increased in DU145 and PC-3 prostate cancer cells after HIF1α-DM transfection (Fig. 1d,e), indicating that HIF1α might regulate the transcription of miR-183-96-182. Consistent with these results, we observed that the expression levels of miR-183-96-182 as well as miR-210, a known HIF1α-regulated miRNA, were all elevated in the prostate of mice treated with dimethyloxalylglycine (DMOG) (Fig. 1f), which stabilized HIF1α expression as a PHD inhibitor (Supplementary Fig. S1a). We also found that the mRNA levels of PHD2 and FIH1 slightly but not significantly decreased after DMOG treatment (Supplementary Fig. S1b). The data from this animal model suggested that HIF1α could also regulate miR-183-96-182 expression *in vivo*. In agreement with this finding, knockdown of HIF1α by three different targeting siRNAs decreased the levels of miR-183-96-182 in PC-3 prostate cancer cells (Fig. 1g,h) under hypoxia. These results suggested that in prostate cancer cells miR-183-96-182 cluster is hypoxia-responsive and is positively regulated by HIF1α.

### HIF1α regulates miR-183-96-182 at transcriptional level

**Figure 2:**
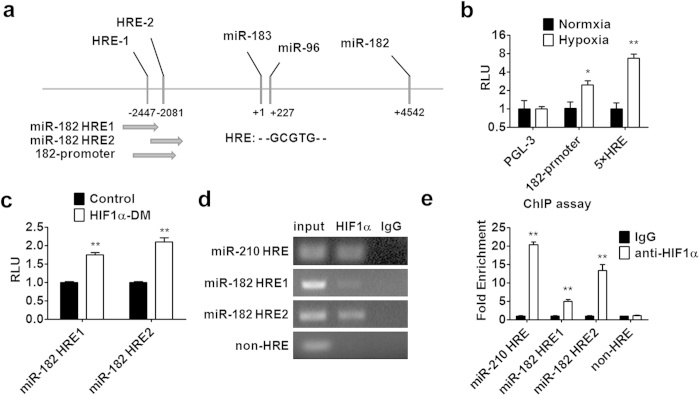
HIF1α regulates miR-183-96-182 expression at transcriptional level. (**a**) The schematic representation of promoter region of human miR-183-96-182 cluster. Two HIF1α response elements (HRE1 and HRE2) are shown. (**b**) The 293T cells were transfected with pGL3 or miR-182 promoter reporter plasmid or 5 × HRE reporter. After 24 hours, the cells were exposed to a hypoxia condition for 24 hours. Then the luciferase activity was determined. (**c**) The 293T cells were **c**o-transfected with HIF1α-DM and miR-182 HRE1 or miR-182 HRE2 reporter plasmids. After 48 hours, luciferase activity was analyzed. (**d**,**e**) ChIP assay was performed by using anti-HIF1α antibody. The elutes were analyzed by using primers for miR-183-96-182 promoter, or miR-210 HRE, or non-HRE region. The quantitative results were obtained by electrophoretic assay or real-time PCR (**d**,**e**). Data are mean ± SEM of three independent experiments. *p < 0.05, **p < 0.01.

HIF1α is a transcriptional factor and regulates target gene transcription by binding to the hypoxia-responsive element (HRE) in the promoter region of its target gene. Two potential HRE sites (designated as miR-182 HRE1 and miR-182 HRE2, respectively) were identified within the upstream region of miR-183-96-182 cluster (named as “182-promoter” in Fig. 2a). A miR-183-96-182 reporter construct with a luciferase was generated. The promoter activity was evaluated in 293T cells under hypoxia condition. As shown in Fig. 2b, hypoxic conditions could increase 182-promoter activity. A reporter containing five repeated HREs (5XHRE)^20^ was used as a positive control. To test which HRE site contributes to the regulation by HIF1α, the reporter containing either HRE1 or HRE2 was examined. As shown in Fig. 2c, transfection of HIF1α-DM into 293T cells could activate both reporters, suggesting that both HRE sites are functional. To determine whether HIF1α could be recruited to the promoter region of miR-183-96-182, chromatin immunoprecipitation (ChIP) assay was performed in PC-3 cells under hypoxic environment as described in Materials and Methods. Consistent with the data from luciferase assay, HIF1α could be recruited to both HRE sites in the miR-183-96-182 promoter (Fig. 2d,e). Notably, more HIF1α protein was recruited to HRE2 site, compared to HRE1 site; however, there is no significant difference between luciferase activities from HRE1- and HRE2-reporter. The sequence of HRE2 is more conserved than that of HRE1 among different species, which might be able to explain this observation (Supplementary Fig. S2). Consistent with a previous report, we also found that HIF1α could be recruited to the HRE site located in the miR-210 promoter^20^. Taken together, our data demonstrated that miR-183-96-182 is a direct target gene of HIF1α.

### miR-182 controls PHD2 and FIH1 levels by directly targeting their 3′-UTR

**Figure 3:**
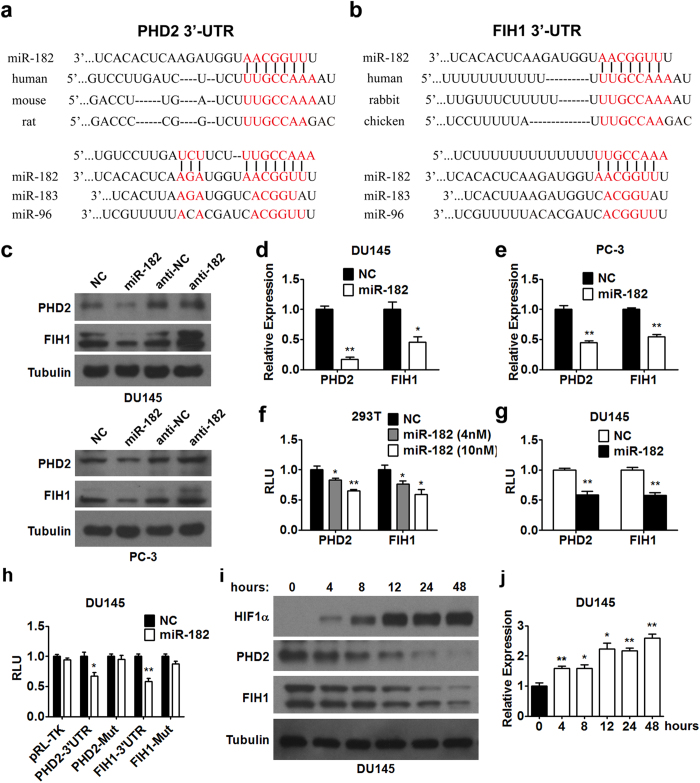
miR-182 regulates PHD2 and FIH1 expression by directly targeting their 3′-UTR. (**a**,**b**) The sequences of predicted and conserved miR-182 binding sites in PHD2 3′-UTR (**a**) and FIH1 3′-UTR (**b**). The binding site is conserved across species. The predicted binding sites for miR-96 and miR-183 are also shown. (**c**) Western blot analysis of PHD2 and FIH1 protein expression in DU145 and PC-3 cells transfected with miR-182 mimics, control oligos (NC), miR-182 antagomir (anti-182), or antagomir control (anti-NC) as indicated. (**d**,**e**) RT-PCR analysis of PHD2 and FIH1 expression in DU145 (**d**) and PC-3 (**e**) cells transfected with miR-182 mimics and control oligos (NC). (**f**) miR-182 significantly inhibits PHD2 and FIH1 3′-UTR reporter luciferase activity in 293T cells. Different amount of miR-182 mimics (4 nM, 10 nM) or control oligos (NC) were co-transfected with reporter plasmids as indicated. (**g**) miR-182 suppresses activity of the reporter containing the 3′-UTR of PHD2 or FIH1 in DU145 prostate cancer cells. (**h**) Luciferase reporter containing wild type or mutant 3′-UTR of PHD2 and FIH1 was co-transfected into DU145 cells with miR-182 or control oligos (NC). 48 hours after transfection, luciferase activity was measured. (**i**,**j**) Cellular hypoxia was induced in DU145 cells for different times as indicated. HIF1α, PHD2, and FIH1 protein levels were determined by western blot analysis (**i**). miR-182 expression was analyzed by RT-PCR (j, n = 3). Data are mean ± SEM of three independent experiments. *p < 0.05, **p < 0.01. Full-length blots are presented in Supplementary Fig. S6.

Since miR-183-96-182 is hypoxia-responsive under the regulation by HIF1α, whether miR-183-96-182 plays regulatory roles in hypoxia adaption or angiogenesis in prostate cancer was further explored. PHD2 and FIH1, two pivotal regulators of HIF1α in hypoxia adaption, were identified as potential targets of miR-182 across species according to TargetScan (top panels in Fig. 3a,b). miR-182 contains more nucleotides complementary to the sequences in the 3′-UTR of PHD2 and FIH1 as compared with miR-96 and miR-183 (bottom panels in Fig. 3a,b). Therefore, we focused on the function of miR-182 in the following study.

To confirm whether PHD2 and FIH1 are target genes of miR-182, the protein expression levels of PHD2 and FIH1 were assessed in DU145 and PC-3 prostate cancer cells transfected with miR-182 mimics. miR-182 overexpression decreased protein (Fig. 3c) and mRNA levels (Fig. 3d,e) of both PHD2 and FIH1; meanwhile, miR-182 inhibition by antagomir (anti-182) increased PHD2 and FIH1 protein expression in these two cell lines (Fig. 3c). We also investigated the effect of miR-182 on the activity of the reporters containing the 3′-UTR of PHD2 and FIH1 in 293T cells. As shown in Fig. 3f, miR-182 mimics transfection repressed the luciferase activity of the two reporters carrying miR-182 regulatory elements found in the 3′-UTR of PHD2 and FIH1, respectively, in a dose-dependent manner. We also observed that miR-182 overexpression by mimic transfection could suppress the PHD2 and FIH1 3′-UTR luciferase reporter activity in DU145 prostate cancer cells (Fig. 3g). As expected, miR-182 overexpression did not have any effect on the activity of the reports with mutations in miR-182 regulatory elements (Fig. 3h and Supplementary Fig. S3a). In agreement with the finding that PHD2 and FIH1 are target genes of miR-182, PHD2 and FIH1 protein levels were decreased gradually, while HIF1α protein levels and miR-182 expression levels were increased in a gradual manner in DU145 and PC-3 cells after cellular hypoxia was induced (Fig. 3i,j, and Supplementary Fig. 3b,c). Together, these results suggested that PHD2 and FIH1 are direct target genes of miR-182.

### miR-182 enhances the expression of HIF1α in prostate cancer cells

**Figure 4:**
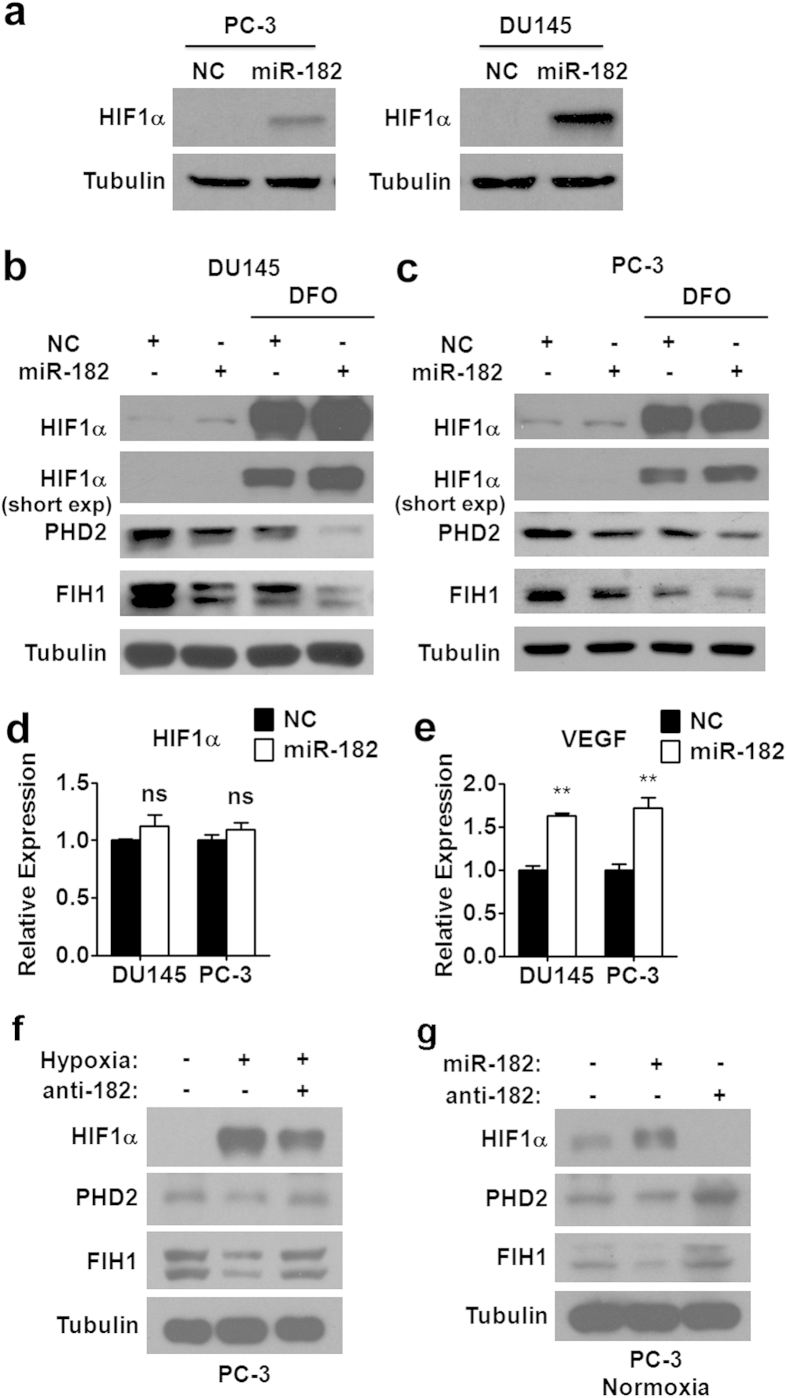
miR-182 enhances the expression levels of HIF1α. (**a**) Western blot analysis of HIF1α protein levels in DU145 and PC-3 cells transfected with miR-182 mimics and control oligos (NC). (**b**,**c**) Western blot analysis of HIF1α, PHD2 and FIH1 protein levels in DU145 (**b**) and PC-3 (**c**) cells transfected with miR-182 mimics and control oligos (NC) in the presence of hypoxia-mimetic DFO. (**d**,**e**) RT-PCR analysis of HIF1α (**d**) and VEGF (**e**) mRNA levels in DU145 and PC-3 cells transfected with miR-182 mimics and control oligos (NC). (**f**) Western analysis of HIF1α, PHD2, and FIH1 protein expression in PC-3 cells transfected with miR-182 antagomir (anti-182) after the induction of cellular hypoxia. (**g**) Western analysis of HIF1α, PHD2, and FIH1 protein expression in PC-3 cells transfected with miR-182 mimics or miR-182 antagomir (anti-182) in a normoxic environment. Data are mean ± SEM of three independent experiments. *p < 0.05, **p < 0.01. Full-length blots are presented in Supplementary Fig. S6.

Since it is known that the stability of HIF1α could be regulated by PHD2 and FIH1, we hypothesized that miR-182, as a upstream regulator of PHD2 and FIH1, could affect HIF1α expression levels. To test our hypothesis, we first examined the effect of miR-182 on HIF1α protein expression under normoxia. As expected, miR-182 overexpression resulted in an elevation of HIF1α protein levels in either DU145 or PC-3 prostate cancer cells (Fig. 4a). We also investigated the effect of miR-182 on HIF1α expression in the presence of hypoxia inducer DFO. As shown in Fig. 4b,c, DFO treatment dramatically increased the HIF1α expression in DU145 and PC-3 prostate cancer cells, which is concomitant with a reduction in protein levels of PHD2 and FIH1. DFO-induced miR-182 expression might partially contribute to the decreased protein levels of PHD2 and FIH1. Consistent with the finding under normoxia, overexpression of miR-182 also could repress the expression of PHD2 and FIH1 in the presence of DFO, which resulted in a further increase in HIF1α protein levels (Fig. 4b,c). To be noted, we did not observe any alteration of HIF1α mRNA levels in DU145 and PC-3 prostate cancer cells overexpressing miR-182 (Fig. 4d), which is consistent with the working model of the regulation of HIF1α by PHD2 and FIH1. We also checked the expression of VEGF, a known HIF1α target gene, in these two prostate cancer cell lines. As expected, the VEGF mRNA expression was increased after miR-182 mimics transfection (Fig. 4e), further suggesting that miR-182 could control HIF1α expression level in prostate cancer cells.

We also took a different approach by using anti-182 to confirm the effect of miR-182 on the expression of PHD2 and FIH1. We found that miR-182 inhibition by antagomir is able to rescue the downregulation of PHD2 and FIH1 under a hypoxic condition, which is accompanied by a downeregulation of HIF1α (Fig. 4f and Supplementary Fig. S4a). Interestingly, we found that anti-182 also could affect the levels of PHD2, FIH1, and HIF1α, under normoxia condition in PC-3 cells, suggesting that the endogenous normoxic miR-182 already plays a role in HIF1α pathway (Fig. 4g and Supplementary Fig. S4b).

In addition, we also tested the effect of miR-182 overexpression and inhibition on PHD2 expression in PC-3 cells transfected with HIF1α-DM, which would upregulate miR-182 expression at transcriptional level (Figs 1 and 2). As shown in Supplementary Fig. S4c, overexpression of miR-182 further reduced the levels of PHD2 in the presence of HIF1α-DM, while inhibition of miR-182 by anti-182 restored the PHD2 expression in the presence of HIF1α-DM. This result further support the notion that miR-182 plays a role in the regulation of PHD2 upon hypoxia or when HIF1α is activated.

### miR-182 promotes prostate tumor angiogenesis and growth

**Figure 5:**
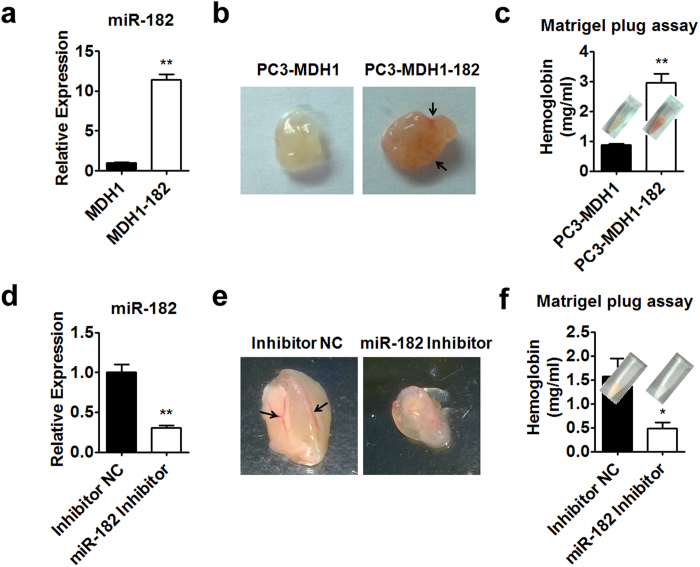
miR-182 positively regulates prostate tumor angiogenesis. (**a**) RT-PCR analysis of miR-182 levels in PC-3 cells infected with MDH1 (PC-3-MDH1) and MDH1-182 retrovirus (PC-3-MDH1-182). (**b**,**c**) *In vivo* matrigel plug assay. Matrigel mixed with PC-3-MDH1 or PC-3-MDH1-182 cells were injected into BALB/cA-nu/nu nude mice. The gross morphology (**b**) and hemoglobin content (**c**) of matrigel plugs were shown. (**d**) RT-PCR analysis of miR-182 levels in PC-3 cells transfected with miR-182 inhibitor or inhibitor negative control (NC). (**e**,**f**) *In vivo* matrigel plug assay. Matrigel mixed with PC-3 cells transfected with miR-182 inhibitor or inhibitor NC were injected into BALB/cA-nu/nu nude mice. The corresponding gross morphology (**e**) and hemoglobin content (**f**) of matrigel plugs were shown. Data are mean ± SEM (n = 6). *p < 0.05, **p < 0.01.

It is known that HIF1 could induce VEGF expression, consequently promote tumor angiogenesis and growth^32^. Above results have shown that miR-182 could enhance the expression of HIF1α and VEGF in prostate cancer cells. Therefore, we hypothesized that miR-182 is able to promote prostate tumor angiogenesis and growth. To test our hypothesis, PC-3 cells were infected with miR-182 retrovirus and sorted. As shown in Fig. 5a, miR-182 retrovirus infection increased the miR-182 levels by more than 10-fold in PC-3 cells. Then, matrigel plug assay was performed to investigate the angiogenesis in miR-182 overexpressing PC-3 xenografts in nude mice. We found that overexpression of miR-182 could increase angiogenesis and hemoglobin content in matrigel plug (Fig. 5b,c). In agreement with this result, knockdown of miR-182 by specific inhibitor reduced angiogenesis and hemoglobin content in matrigel plugs of PC-3 xenografts (Fig. 5d–f). These *in vivo* results demonstrated that miR-182 is a positive regulator of angiogenesis.

**Figure 6:**
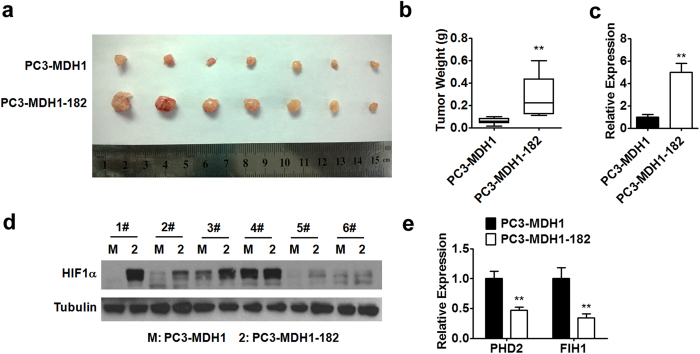
miR-182 promotes tumor growth by targeting HIF1α pathway. (**a**–**c**) PC-3-MDH1 or PC-3-MDH1-182 cells were injected subcutaneously into nude mice (n = 6). The overexpression of miR-182 in xenografts was confirmed by RT-PCR analysis (**c**). Tumors were photographed (**a**) and weighed (**b**) as indicated. (**d**) Western blot analysis of HIF1α protein levels in PC-3-MDH1 and PC-3-MDH1-182 xenografts. (**e**) PHD2 and FIH1 mRNA levels in xenografts were analyzed by RT-PCR. Data are mean ± SEM. **p < 0.01. Full-length blots are presented in Supplementary Fig. S6.

Tumor size and weight of PC-3 xenografts were also measured. Consistent with previous reports^28^, tumors derived from PC-3 prostate cancer cells overexpressing miR-182 had larger size and heavier weight as compared to the control group (Fig. 6a–c). If our hypothesis is correct, we would expect to see the altered expression of PHD2 and FIH1 as well as HIF1α in the tumors derived from miR-182 overexpressing prostate cancer cells. Indeed, we observed that the protein levels of HIF1α were increased in these miR-182 overexpressing xenografts (Fig. 6d). Moreover, the mRNA expression of PHD2 and FIH1 was decreased in miR-182 overexpressing tumors (Fig. 6e).

### miR-183-96-182 and HIF1α expression were upregulated in PTEN^PC−/−^ mice

**Figure 7:**
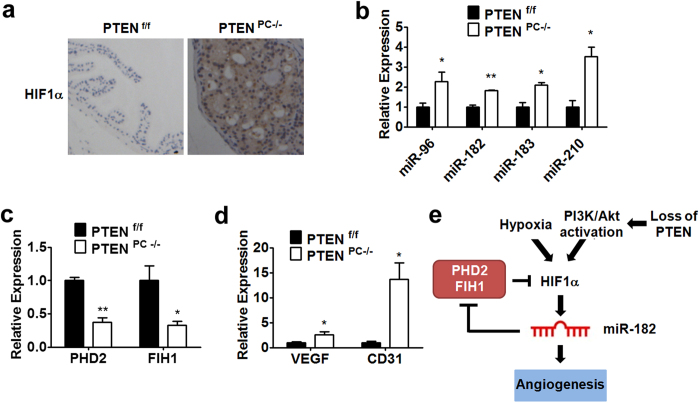
miR-183-96-182 and HIF1α expression were upregulated in PTEN^PC−/−^ mice. (**a**) Immunohistochemical staining of HIF1α. Theprostate tissues were stained by HIF1α antibody. (**b**) RT-PCR analysis of miR-183-96-182 in prostate tissues from PTEN^PC−/−^ mice (n = 3). The expression level of miR-210 was also determined. (**c**,**d**) The expression of PHD2, FIH1 (**c**) VEGF and CD31 (**d**) in prostate tissues was determined by RT-PCR. Data are mean ± SEM (n = 3). *p < 0.05, **p < 0.01. (**e**) The model of HIF1α-miR-182 positive feedback loop pathway. Decrease in oxygen level or loss of PTEN, which leads to PI3K/Akt activation, in prostate cancer increases HIF1α protein level, which upregulates miR-182 expression at transcriptional level. Increased miR-182 expression represses PHD2 and FIH1 expression by directly targeting their 3′-UTR, which results in an accumulation of HIF1α protein. This positive feedback loop maintains hyperactive HIF1α signaling in prostate tumors, which facilitates angiogenesis and tumor growth in hypoxic environment.

It has been reported that HIF1α expression could be regulated by PI3K/PTEN/Akt pathway in cells including prostate cancer cells^15^,^33^,^34^. However, whether HIF1α expression was upregulated in PTEN^PC−/−^ mice, a mouse model of spontaneous prostate cancer^35^, is not clear. In this study, the loss of PTEN function was confirmed by the activation of Akt in the PTEN^PC−/−^ prostates (Supplementary Fig. S5a). Overexpressed HIF1α proteins were detected in the PTEN^PC−/−^ prostates by immunohistochemical staining (Fig. 7a). As expected, the levels of miR-182 as well as miR-183 and miR-96 were all significantly upregulated in the prostate of PTEN^PC−/−^ mice (Fig. 7b). Moreover, the expression of miR-210, another hypoxia inducible miRNA, also increased (Fig. 7b). We also observed that the mRNA expression of PHD2 and FIH1 was reduced, while the mRNA expression of VEGF and CD31, another HIF1α-regulated gene, was increased in the prostate of PTEN^PC−/−^ mice (Fig. 7c,d). Thus, the positive correlation between miR-182 level and HIF1α level and the negative correlation between the level of miR-182 and the level of PHD2 and FIH1 further support the hypothesis that increased level of HIF1α could lead to the elevation of miR-182 level and the elevated miR-182 might in turn stabilize HIF1α by targeting PHD2 and FIH1.

We also performed the experiment using PI3K inhibitor LY294002 to see whether PI3K/Akt signaling are required for the HIF1α and miR-182 upregulation. As shown in Supplementary Fig. S5b and c, inhibition of PI3K by LY294002 significantly reduced the expression of miR-182 and HIF1α upon DFO-induced hypoxia, indicating that the constitutive activation of PI3K/Akt signaling due to PTEN loss contributes to the elevation of miR-182 and HIF1α.

## Discussion

Prostate cancer is one of the most popular and aggressive cancers in men worldwide. Emerging evidence indicated that both genetic and environmental factors are major factors for prostate cancer development and progression. However, the molecular mechanisms underlying the tumorigenesis of prostate cancer are still poorly understood. Hypoxia is a hallmark of cancer. It is well accepted that adaptation to hypoxic stress is critical for cancer cell survival. Transcription factor HIF1α plays critical roles in cellular response to hypoxia. Modulation of HIF1α levels is a rate-limiting step in hypoxic response. It is known that the expression level of HIF1α is regulated at multiple levels^10^,^11^. Here we identified a novel regulatory mechanism that hypoxia-responsive and HIF1α-regulated miR-182 acts as a positive regulator of HIF1α signaling by targeting negative regulators PHD2 and FIH1 in prostate cancer cells. This miR-182-HIF1α positive feedback loop might facilitate the angiogenesis and tumor growth in prostate cancer.

miRNAs are small regulatory RNA molecules of 21–23 nucleotides in length which suppress target gene at posttranscriptional level. It is known that miR-182 is upregulated in various cancer types including prostate cancer^27^,^36^,^37^,^38^. In prostate cancer, miR-182 could affect zinc homeostasis, promote cell proliferation and invasion, and induce mesenchymal to epithelial transition and growth factor independent growth by targeting different genes. Nevertheless, the transcriptional regulation of miR-182 as well as its role in angiogenesis during prostate cancer progression is not clear. Here we demonstrated that miR-182 is a direct target gene of HIF1α and is upregulated in response to hypoxia. Our data also suggested that miR-182 could facilitate HIF1α-controlled angiogenesis through targeting PHD2 and FIH1, two negative regulators of HIF1α signaling. Thus miR-182-induced angiogenesis enables the prostate cancer cells to obtain enormous oxygen and nutrient, and finally results in outgrowth of prostate tumor.

HIF1α is unstable under normoxia due to ubiquitin-mediated proteasomal degradation, but is stabilized in hypoxic conditions. The degradation of HIF1α is mediated through hydroxylation by PHD. The activity of HIF1 can also be inhibited by FIH1, which also hydroxylates HIF1α. It is known that HIF1α could induce the expression of PHD3^31^, but the expression of PHD2 and FIH1 under hypoxic condition is very complicated in different cell lines, especially in prostate cancer cells. Here, we demonstrated that the expression of PHD2 and FIH1 were decreased under hypoxic condition in PC-3 and DU145 cells, while inhibition of miR-182 could rescue it, suggesting that the downregulation of PHD2 and FIH1 upon hypoxia stress is partially due to the increased level of miR-182 in prostate cancer cells. Notably, we did not detect a significant reduction of the mRNA levels of PHD2 and FIH1 in the prostate of mice after DMOG treatment, indicating that other compensatory mechanisms existed in this mouse model. Because of the important role of HIF1 in cancer, investigation of the regulation of HIF1α might provide novel therapeutic targets. miRNA is a new class of regulators which play critical roles in growth, development, differentiation, and metabolism. miRNA not only is important for physiological but pathological condition. Here we showed that miR-182 provides another level of complexity in the regulation of HIF1α function. Currently, a growing number of anticancer agents have been developed to inhibit HIF1 activity including: 1) inhibition of HIF1α at mRNA level or at protein level, 2) suppression of HIF1 DNA-binding ability, and 3) inhibition of HIF1-mediated gene transcription. Our finding of the regulation of HIF1α by miR-182 provides another approach to suppress HIF1α action. Our data also indicated that miR-182 inhibitor could be tested for prostate cancer treatment.

Alterations of multiple tumor suppressor genes were found in prostate cancer. Mutations in PTEN or others in PI3K signaling pathway are the most common genetic alterations reported in human primary and metastatic prostate cancer^34^,^35^. PTEN and PI3K signaling pathway is able to regulate HIF1α expression and activity^15^,^33^,^34^. We here found that the HIF1α expression levels were increased in PTEN^PC−/−^ mice further supporting the notion that PI3K/PTEN signaling could target HIF1α expression. We also observed that miR-182 expression was increased and its target genes, PHD2 and FIH1, were downregulated in PTEN^PC−/−^ mice. Based on these findings, we proposed that the increased expression of HIF1α stimulates miR-182 expression and the elevated miR-182 expression in turn contribute to the accumulation of HIF1α in the prostate of PTEN^PC−/−^ mice. The miR-182-HIF1α positive feedback loop results in hyperactivation of HIF1α signaling and increased angiogenesis in tumor growth, thereby helping the cancer cells survive under hypoxic environment.

Taken together, we proposed that miR-182 is activated by hypoxia and HIF1α, and inhibits negative regulators (PHD2 and FIH1) of the HIF1α signaling pathway (Fig. 7e). In this scenario, miR-182 would promote the irreversible activation of the HIF1α pathway and thereby the stable switching of the cellular state for tumor growth and angiogenesis under the hypoxic condition. miR-182 as a potential drug target for prostate cancer is also suggested.

## Materials and Methods

### Cell culture, transfection and luciferase assay

PC-3 cells and DU-145 cells were cultured in RPMI-1640 (Invitrogen Inc.) medium supplemented with 10% fetal bovine serum, 2 mM L-glutamine, 100 units/mL penicillin, and 100 mg/mL streptomycin. 293T cells were cultured in Dulbecco’s Modified Eagle Medium (Invitrogen Inc.) supplemented with 10% fetal bovine serum, 100 units/mL penicillin, and 100 mg/mL streptomycin. The cells were originally obtained from the American Type Culture Collection. Cultures were maintained at 37 °C in a humidified incubator, 21% O_2_, 5% CO_2_ atmosphere. Cellular hypoxia was induced by treatment with DFO (100 uM, Sigma-Aldrich). When indicated, cells were incubated in a hypoxic chamber with 1% O_2_, 5% CO_2_ and 94% N_2_. Transfection was performed using Lipofectamine 2000 (Invitrogen Inc.). Cells were harvested 48 hours after transfection. Luciferase assays were performed by using the Dual-Luciferase Reporter Assay System (Promega) following the manufacturer’s instructions. Luciferase activity was measured on a luminometer (Berthold Technologies).

### Plasmid and RNA oligonucleotide

The miR-182 precusors was amplified by PCR using genomic DNA as a template, and the PCR product was cloned into MDH1-IRES-GFP vector (Addgene) to generate miRNA expression plasmids. The pcDNA3.1- HIF1α-DM was gifted from Dr. J Fang (SIBS, CAS, CHINA). For construction of luciferase reporter plasmids, the 3′ UTR fragment of PHD2 and FIH1 were amplified from human cDNA and inserted into pRL-TK vector. To generate mutations in miR-182 regulatory elements, KOD-Plus mutagenesis kit (Toyobo) was used according to the manufacturer’s instruction. Enhancer or possible promoter sequences of miR-183/96/182 cluster containing two HRE sites were amplified by using human genomic DNA and inserted into pGL3 vector or pTK109-luc vector. GMR-miR^TM^ microRNA double-stranded mimics for miR-182, inhibitor for miR-182, and miRNA miR-Down^TM^ antagomir (anti-182) were obtained from Genepharma. The control siRNA and siHIF1α RNA oligos were purchased from Genepharma. MicroRNA and siRNA information will be found in the Supplementary Table 1.

### Real time-PCR

Trizol Reagent was used to isolate total RNA from frozen tissue samples or cell lines according to the manufacturer’s instructions. The extraction of small RNA (≤200 nt) was performed as previously described^39^. First strand cDNA was synthesized from total RNA with PrimeScript RT reagent Kit (TaKaRa). Small RNA was reverse-transcribed by using poly A polymerase RT system, which was performed according to instructions. Real-time PCR was performed on an ABI7900 Real-Time PCR System (Applied Biosystems). The list of primers used was provided in the Supplementary Table 2. The expression of miRNAs in the cells and tissues was normalized to U6 levels, while the expression of other mRNAs was normalized by the expression of 18S rRNA as a reference housekeeping gene.

### Western Blot analysis

Cultured cells and frozen tissue samples were lysed in RIPA lysis buffer (50 mM Tris-HCl, pH 7.5, 150 mM NaCl, 1.0 mM EDTA, 0.1% SDS, 1% Sodium deoxycholate, and 1% Triton X-100), which containing Protease Inhibitor Cocktail and Phosphatase Inhibitor Cocktail (Sigma-Aldrich). The protein concentration was determined using a BCA protein assay kit (Thermo Fisher). Protein lysates were resolved on 10% SDS-PAGE gels using standard procedures. Anti-HIF1α (BD Biosciences), anti-PHD2 (Santa Cruz), anti-FIH1 (Santa Cruz), anti-p-Akt (Cell Signaling), anti-Akt (Cell Signaling), anti-actin (Sigma-Aldrich), and anti-α-tubulin (Sigma-Aldrich) antibodies were used for western blot analysis. Uncropped images of western blots are presented in Supplementary Fig. S6.

### Retrovirus preparation and infection

The retrovirus was conducted as described. In brief, supernatant contained high-titer retrovirus was generated by co-transfection of a retrovirus vector MDH1 and the gag/pol, VSVG viral packaging construct into 293T cells. PC-3 cells were infected with retrovirus that encodes the miR-182. Infected cells were analyzed and sorted by a FACS scan (Beckman Coulter) with a standard excitation wavelength of 488 nm. Overexpression of miR-182 in infected PC-3 cells was confirmed by real-time PCR.

### Chromatin immunoprecipitation (ChIP) assay

ChIP assays were performed using the EZ Magna ChIP kit (Millipore) according to the manufacturer’s protocol. PC-3 cells were incubated in a hypoxic chamber (1% O_2_) 24 hours before harvest. Anti-HIF1α (Abcam) was used to precipitate DNA fragments. PCR was performed to analyze HIF1α binding site. miR-210 HRE was used as a positive control. A primer set for non-HRE region was used as a negative control. ChIP primers will be found in the Supplementary Table 3.

### Animal experiments

Animals were maintained and experiments were performed according to protocols approved by the Animal Care and Use Committees of Institute for Nutritional Sciences (permit number: 2011-AN-14). Prostate tissues from PTEN^PC−/−^ mice (36–40 weeks old) and age-matched C57BL/6 mice were used for real-time PCR or immunohistochemistry analysis as described. Regarding the DMOG administration, C57BL/6 mice were injected ip with 20 mg DMOG per mouse or with PBS solution on day 1 and day 3. On day 4 (24 h after last injection), the prostate tissues were harvested for expression analysis. For *in vivo* Matrigel plug assay, briefly, BALB/cA-nu/nu male nude mice (4 weeks old, from Shanghai Experimental Animal Center) were injected subcutaneously with PC-3 cells (3 × 10^6^) in a mixture (1:2 v/v) of PBS and Matrigel (BD Biosciences). On day 12–14, the mice were sacrificed, and plugs were harvested for hemoglobin assay. Drabkin’s assay (Sigma-Aldrich) was used for hemoglobin quantification^40^,^41^. The experiments of *in vivo* xenograft growth assay were conducted as described. In brief, PC-3 cells (2 × 10^6^) were mixed with Matrigel (1:1 v/v). The mixture (200 ul) was injected subcutaneously into either flank sides of the same BALB/cA-nu/nu male nude mouse (4–5 weeks old). Tumors were harvested 3–4 weeks after injection.

### Immunohistochemical staining

Ventral prostate of PTEN^f/f^ and PTEN^PC−/−^ mice were fixed and processed by paraffin-embedded method. Mouse polyclonal antibody against HIF1α (Abcam) and rabbit monoclonal antibody against p-Akt (Cell signaling) were used. The sections were counterstained with hematoxylin.

### Equipment and settings

Detection of western blot images was performed using Chemi-Doc Imaging System (Bio-Rad) and the software Quantity One. All Digital images were visualized at room temperature using a microscope (Olympus BX61), a cooled CCD camera (QICAM Fast, QImaging Corporation.) and the software package Q-Capture (version 2.9.11, QImaging Corporation) with UPlanApo 20×/0.70 (Olympus) objective lens.

### Statistical analysis

For all data are represented as means ± SEM. Student’s t-test was used to analyze the difference between two groups. The significance is presented as *p < 0.05, **p < 0.01 and ***p < 0.005, and non-significant differences are presented as ns. GraphPad Prism 5.0 (GraphPad Software) was used for all statistical analysis.

## Additional Information

**How to cite this article**: Li, Y. *et al.* Hypoxia-inducible miR-182 enhances HIF1α signaling via targeting PHD2 and FIH1 in prostate cancer. *Sci. Rep.* **5**, 12495; doi: 10.1038/srep12495 (2015).

## Supplementary Material

Supplementary Information

## References

[b1] Bertout J. A., Patel S. A. & Simon M. C. The impact of O2 availability on human cancer. Nat. Rev. Cancer 8, 967–975 (2008). 18987634 10.1038/nrc2540 PMC3140692

[b2] Denko N. C. *et al.* Investigating hypoxic tumor physiology through gene expression patterns. Oncogene 22, 5907–5914 (2003). 12947397 10.1038/sj.onc.1206703

[b3] Hockel M. & Vaupel P. Tumor hypoxia: Definitions and current clinical, biologic, and molecular aspects. J. Natl. Cancer Inst. 93, 266–276 (2001). 11181773 10.1093/jnci/93.4.266

[b4] Nanni S. *et al.* Endothelial NOS, estrogen receptor beta, and HIFs cooperate in the activation of a prognostic transcriptional pattern in aggressive human prostate cancer. J. Clin. Invest. 119, 1093–1108 (2009). 19363294 10.1172/JCI35079 PMC2673846

[b5] Movsas B. *et al.* Increasing levels of hypoxia in prostate carcinoma correlate significantly with increasing clinical stage and patient age: an Eppendorf pO(2) study. Cancer 89, 2018–2024 (2000). 11064360 10.1002/1097-0142(20001101)89:9<2018::aid-cncr19>3.3.co;2-p

[b6] Bostwick D. G. & Iczkowski K. A. Microvessel density in prostate cancer: prognostic and therapeutic utility. Semin. Urol. Oncol. 16, 118–123 (1998). 9741415

[b7] Pallares J. *et al.* Study of microvessel density and the expression of the angiogenic factors VEGF, bFGF and the receptors Flt-1 and FLK-1 in benign, premalignant and malignant prostate tissues. Histol. Histopathol. 21, 857–865 (2006). 16691538 10.14670/HH-21.857

[b8] Marignol L., Coffey M., Lawler M. & Hollywood D. Hypoxia in prostate cancer: a powerful shield against tumour destruction? Cancer Treat. Rev. 34, 313–327 (2008). 18334284 10.1016/j.ctrv.2008.01.006

[b9] Semenza G. L. Hypoxia-inducible factor 1 (HIF-1) pathway. Sci. STKE. 2007, cm8 (2007). 17925579 10.1126/stke.4072007cm8

[b10] Bruick R. K. & McKnight S. L. A conserved family of prolyl-4-hydroxylases that modify HIF. Science 294, 1337–1340 (2001). 11598268 10.1126/science.1066373

[b11] Mahon P. C., Hirota K. & Semenza G. L. FIH-1: a novel protein that interacts with HIF-1alpha and VHL to mediate repression of HIF-1 transcriptional activity. Genes Dev. 15, 2675–2686 (2001). 11641274 10.1101/gad.924501 PMC312814

[b12] Metzen E. *et al.* Regulation of the prolyl hydroxylase domain protein 2 (phd2/egln-1) gene: identification of a functional hypoxia-responsive element. Biochem J. 387, 711–717 (2005). 15563275 10.1042/BJ20041736 PMC1135001

[b13] Bardos J. I. & Ashcroft M. Hypoxia-inducible factor-1 and oncogenic signalling. Bioessays 26, 262–269 (2004). 14988927 10.1002/bies.20002

[b14] Gottlieb E. & Tomlinson I. P. Mitochondrial tumour suppressors: a genetic and biochemical update. Nat. Rev. Cancer 5, 857–866 (2005). 16327764 10.1038/nrc1737

[b15] Semenza G. L. Targeting HIF-1 for cancer therapy. Nat. Rev. Cancer 3, 721–732 (2003). 13130303 10.1038/nrc1187

[b16] Zhong H. *et al.* Overexpression of hypoxia-inducible factor 1alpha in common human cancers and their metastases. Cancer Res. 59, 5830–5835 (1999). 10582706

[b17] Zhong H., Semenza G. L., Simons J. W. & De Marzo A. M. Up-regulation of hypoxia-inducible factor 1alpha is an early event in prostate carcinogenesis. Cancer Detect. Prev. 28, 88–93 (2004). 15068831 10.1016/j.cdp.2003.12.009

[b18] Ranasinghe W. K. *et al.* The effects of nonspecific HIF1alpha inhibitors on development of castrate resistance and metastases in prostate cancer. Cancer Med. 3, 245–251 (2014). 24464861 10.1002/cam4.189 PMC3987074

[b19] Stefani G. & Slack F. J. Small non-coding RNAs in animal development. Nat. Rev. Mol. Cell Biol. 9, 219–230 (2008). 18270516 10.1038/nrm2347

[b20] Huang X. *et al.* Hypoxia-inducible mir-210 regulates normoxic gene expression involved in tumor initiation. Mol. Cell 35, 856–867 (2009). 19782034 10.1016/j.molcel.2009.09.006 PMC2782615

[b21] Kelly T. J., Souza A. L., Clish C. B. & Puigserver P. A hypoxia-induced positive feedback loop promotes hypoxia-inducible factor 1alpha stability through miR-210 suppression of glycerol-3-phosphate dehydrogenase 1-like. Mol. Cell. Biol. 31, 2696–2706 (2011). 21555452 10.1128/MCB.01242-10 PMC3133367

[b22] Wang H. *et al.* Negative regulation of Hif1a expression and TH17 differentiation by the hypoxia-regulated microRNA miR-210. Nat. Immunol. 15, 393–401 (2014). 24608041 10.1038/ni.2846 PMC3996831

[b23] Liu C. J. *et al.* miR-31 ablates expression of the HIF regulatory factor FIH to activate the HIF pathway in head and neck carcinoma. Cancer Res. 70, 1635–1644 (2010). 20145132 10.1158/0008-5472.CAN-09-2291

[b24] Ghosh G. *et al.* Hypoxia-induced microRNA-424 expression in human endothelial cells regulates HIF-alpha isoforms and promotes angiogenesis. J. Clin. Invest. 120, 4141–4154 (2010). 20972335 10.1172/JCI42980 PMC2964978

[b25] Chen Z. *et al.* Hypoxia-responsive miRNAs target argonaute 1 to promote angiogenesis. J. Clin. Invest. 123, 1057–1067 (2013). 23426184 10.1172/JCI65344 PMC3582133

[b26] Schaefer A. *et al.* Diagnostic and prognostic implications of microRNA profiling in prostate carcinoma. Int. J. Cancer 126, 1166–1176 (2010). 19676045 10.1002/ijc.24827

[b27] Mihelich B. L. *et al.* miR-183-96-182 cluster is overexpressed in prostate tissue and regulates zinc homeostasis in prostate cells. J. Biol. Chem. 286, 44503–44511 (2011). 22045813 10.1074/jbc.M111.262915 PMC3247959

[b28] Hirata H. *et al.* MicroRNA-182-5p promotes cell invasion and proliferation by down regulating FOXF2, RECK and MTSS1 genes in human prostate cancer. PLoS One 8, e55502 (2013). 23383207 10.1371/journal.pone.0055502 PMC3559583

[b29] Liu R., Li J., Teng Z., Zhang Z. & Xu Y. Overexpressed microRNA-182 promotes proliferation and invasion in prostate cancer PC-3 cells by down-regulating N-myc downstream regulated gene 1 (NDRG1). PLoS One 8, e68982 (2013). 23874837 10.1371/journal.pone.0068982 PMC3712934

[b30] Qu Y. *et al.* MiR-182 and miR-203 induce mesenchymal to epithelial transition and self-sufficiency of growth signals via repressing SNAI2 in prostate cells. Int. J. Cancer 133, 544–555 (2013). 23354685 10.1002/ijc.28056

[b31] Appelhoff R. J. *et al.* Differential function of the prolyl hydroxylases PHD1, PHD2, and PHD3 in the regulation of hypoxia-inducible factor. J. Biol. Chem. 279, 38458–38465 (2004). 15247232 10.1074/jbc.M406026200

[b32] Ferrara N. & Davis-Smyth T. The biology of vascular endothelial growth factor. Endocr. Rev. 18, 4–25 (1997). 9034784 10.1210/edrv.18.1.0287

[b33] Zundel W. *et al.* Loss of PTEN facilitates HIF-1-mediated gene expression. Genes Dev. 14, 391–396 (2000). 10691731 PMC316386

[b34] Zhong H. *et al.* Modulation of hypoxia-inducible factor 1alpha expression by the epidermal growth factor/phosphatidylinositol 3-kinase/PTEN/AKT/FRAP pathway in human prostate cancer cells: implications for tumor angiogenesis and therapeutics. Cancer Res. 60, 1541–1545 (2000). 10749120

[b35] Wang S. *et al.* Prostate-specific deletion of the murine Pten tumor suppressor gene leads to metastatic prostate cancer. Cancer Cell 4, 209–221 (2003). 14522255 10.1016/s1535-6108(03)00215-0

[b36] Segura M. F. *et al.* Aberrant miR-182 expression promotes melanoma metastasis by repressing FOXO3 and microphthalmia-associated transcription factor. Proc. Natl. Acad. Sci. USA. 106, 1814–1819 (2009). 19188590 10.1073/pnas.0808263106 PMC2634798

[b37] Hirata H. *et al.* Oncogenic miRNA-182-5p targets Smad4 and RECK in human bladder cancer. PLoS One 7, e51056 (2012). 23226455 10.1371/journal.pone.0051056 PMC3511415

[b38] Lei R. *et al.* Suppression of MIM by microRNA-182 activates RhoA and promotes breast cancer metastasis. Oncogene 33, 1287–1296 (2014). 23474751 10.1038/onc.2013.65

[b39] Zhang D. *et al.* Thyroid hormone regulates muscle fiber type conversion via miR-133a1. J. Cell Biol. 207, 753–766 (2014). 25512392 10.1083/jcb.201406068 PMC4274265

[b40] Shi Z. M. *et al.* MiR-128 inhibits tumor growth and angiogenesis by targeting p70S6K1. PLoS One 7, e32709 (2012). 22442669 10.1371/journal.pone.0032709 PMC3307714

[b41] Sun H. *et al.* TRAF6 upregulates expression of HIF-1alpha and promotes tumor angiogenesis. Cancer Res. 73, 4950–4959 (2013). 23722539 10.1158/0008-5472.CAN-13-0370

