# Supplementary material for: Hypoxia-inducible miR-182 enhances HIF1α signaling via targeting PHD2 and FIH1 in prostate cancer
Source: Sci Rep. 2015 Jul 24;5:12495. doi: 10.1038/srep12495 (PMC4513346; doi:10.1038/srep12495)
Supplement: Supplementary Information [file srep12495-s1.pdf]

Hypoxia-inducible miR-182 enhances HIF1α signaling via targeting PHD2 and FIH1 in prostate cancer

Yan Li[1], Duo Zhang[1], Xiaoyun Wang[1], Xuan Yao[1], Cheng Ye[1], Shengjie Zhang[1], Hui Wang[1], Cunjie Chang[2], Hongfeng Xia[1], Yu-cheng Wang[3,4], Jing Fang[1,5], Jun Yan[¶2], Hao Ying[¶1,4,5]

[1]Key Laboratory of Food Safety Research, Institute for Nutritional Sciences, Shanghai Institutes for Biological Sciences, University of Chinese Academy of Sciences, Chinese Academy of Sciences, Shanghai 200031, China; [2]Model Animal Research Center, and MOE Key Laboratory of Model Animals for Disease Study, Nanjing University, Nanjing 210061, China; [3]Department of Nutrition, Shanghai Xuhui Central Hospital, Shanghai 200031, China; [4]Clinical Research Center of Institute for Nutritional Sciences, Shanghai Institutes for Biological Sciences, Chinese Academy of Sciences, Shanghai 200031, China; [5]Key Laboratory of Food Safety Risk Assessment, Ministry of Health, Beijing, China.

[¶]Corresponding author: Hao Ying, Institute for Nutritional Sciences, SIBS, CAS, 320 Yueyang Road, Shanghai 200031, China, Phone: 86-21-54920247, Fax: 86-21-54920291, E-mail: yinghao@sibs.ac.cn; Jun Yan, Model Animal Research Center, and MOE Key Laboratory of Model Animals for Disease Study, Nanjing University, Nanjing 210061, China, Phone: 86-25-58641535, Fax: 86-25-58641500, E-mail: yanjun@nicemice.cn

Supplementary Table 1. miRNA/siRNAs and oligonucleotide sequences

miRNA/siRNA	Sense Sequence (5'→3')	Antisense Sequence (5'→3')
miR-182	UUUGGCAAUGGUAGAACUCACACU	AGUGUGAGUUCUACCAUUGCCAAA
N.C.	UUCUCCGAACGUGUCACGU	ACGUGACACGUUCGGAGAA
miR-182 Inhibitor	AGUGUGAGUUCUACCAUUGCCAAA	
Inhibitor NC	CAGUACUUUUGUGUAGUACAA	
siHIF1α-854	AAGUCUGCAACAUGGAAGGUtt	ACCUUCCAUGUUGCAGACUUtt
siHIF1α-1035	GACACAGCCUGGAUAUGAAtt	UUCAUAUCCAGGCUGUGUCtt
siHIF1α-1941	AGAACCCAUUUUCUACUCAGtt	CUGAGUAGAAAAUGGGUUCUtt

Supplementary Table 2. Primer sequences for RT-PCR

gene	Sense Sequence (5'→3')	Antisense Sequence (5'→3')
miR-182	TTTGGCAATGGTAGAACTCA	
miR-96	TTTGGCACTAGCACATTTTTGC	
miR-183	TATGGCACTGGTAGAATTCACT	
miR-210	CTGTGCGTGTGACAGCGGCT	
U6	ATTCGTGAAGCGTTCCATAT	
Uni-primer	CGAATTCTAGAGCTCGAGGCAGG	
18S	ACCGCAGCTAGGAATAATGGA	CAAATGCTTTCGCTCTGGTC
PHD2	GTGCCGTGCATGAACAAGC	CAGGTGATCTTATCGCCTCGG
FIH1	GAGTGCCTCTACCCATACCCT	TCGTAGTCGGGATTGTCAAAGT
VEGF	AGGGCAGAATCATCACGAAGT	AGGGTCTCGATTGGATGGCA
HIF1α	CTGATCATCTGACCAAAACTC	GTTTCAACCCAGACATATCCAC
mCD31	CTCATTGCGGTGGTTGTCAT	TTCTGTTT-GGCCTTGGCTTT
mVEGF	CTGCCGTCCGATTGAGACC	CCCCTCCTTGTACCACTGTC
mPHD2	AGCTGGTCAGCCAGAAGAGT	GCCCTCGATCCAGGTGATCT
mFIH1	CATTGGCATGGAAGGAAATGTG	GACAGGGTATGGATAGAGGCA

Supplementary Table 3. Primer sequences for ChIP assay

Name	Sense Sequence (5’→3’)	Antisense Sequence (5’→3’)
miR-182 HRE1	GCTTTTCGAGAGAGCTGGGA	TCGGCTGGGCCAGGTTCCTC
miR-182 HRE2	TCTGTTCTCCAGCTCTAAGG	ATTCTTCTTTTGGGGACAGC
miR-210 HRE	GCAGACGTGCAGAAAAGAACG	CCGTATGCAAATGACCTGGTC
non-HRE	GCCTTCAGGTGGAGATAGGAGA	TTCAAGGAGACAGGGACAGTGC

Supplementary Fig. S1

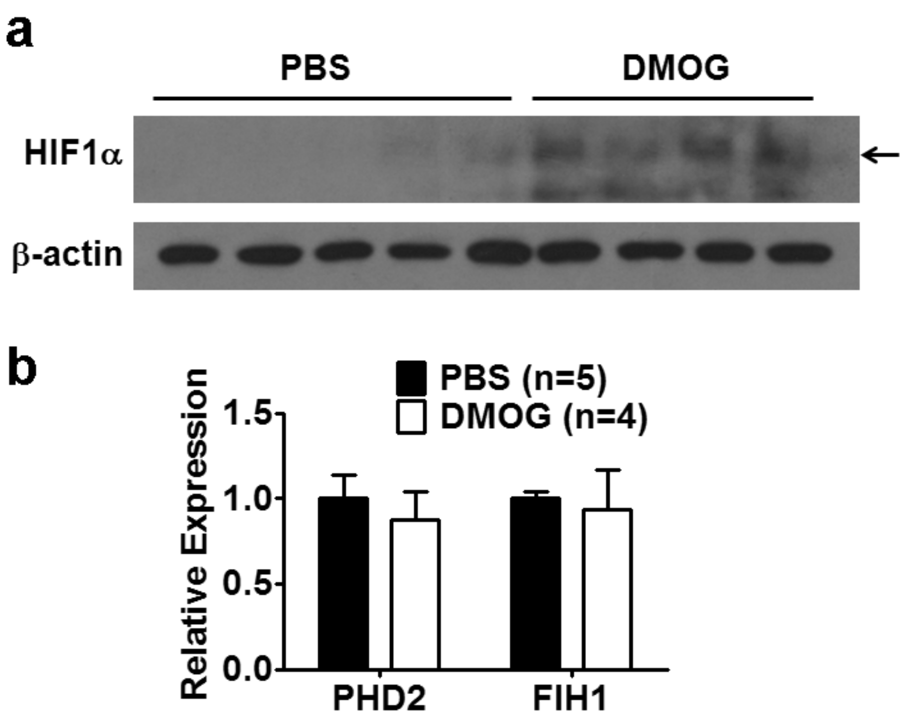

Supplementary Figure. S1.
C57BL/6 mice were treated with DMOG or vehicle as described in Materials and Methods. The prostate tissues were harvested and the HIF1α protein expression (a) and PHD2 and FIH1 miRNA expression (b) were determined by western blot analysis and RT-PCR, respectively (n=4-5). Full-length blots are presented in Supplementary Fig. S6.

Supplementary Fig. S2

HRE1 **HRE2**

human AGCGGGCGTGGCACC------GTGCTGCGTGTTC
chimpanzee AGCGGGCGTGGCACC------GTGCTGCGTGTTC
monkey AGCAGGCCTGGCATC-------GTGCTGCGTGTTC

Supplementary Figure. S2.
Sequence alignment of miR-182 promoter region containing hypoxia-reponsive elements (HREs) from different species as indicated. The HRE1 and HRE2 are shown in red.

Supplementary Fig. S3

a

PHD2-3’UTR: 5’...GUCCUUGAUC----U--UCUUUGCCAAAAU...3’
MRE-mutant: 5’...GUCCUUGAUC----U--UCUAACGGUUUAU...3’

FIH1-3’UTR: 5’...UUUUUUUUUUU----------UUUGCCAAAAU...3’
MRE-mutant: 5’...UUUUUUUUUUU----------UAACGGUUUAU...3’

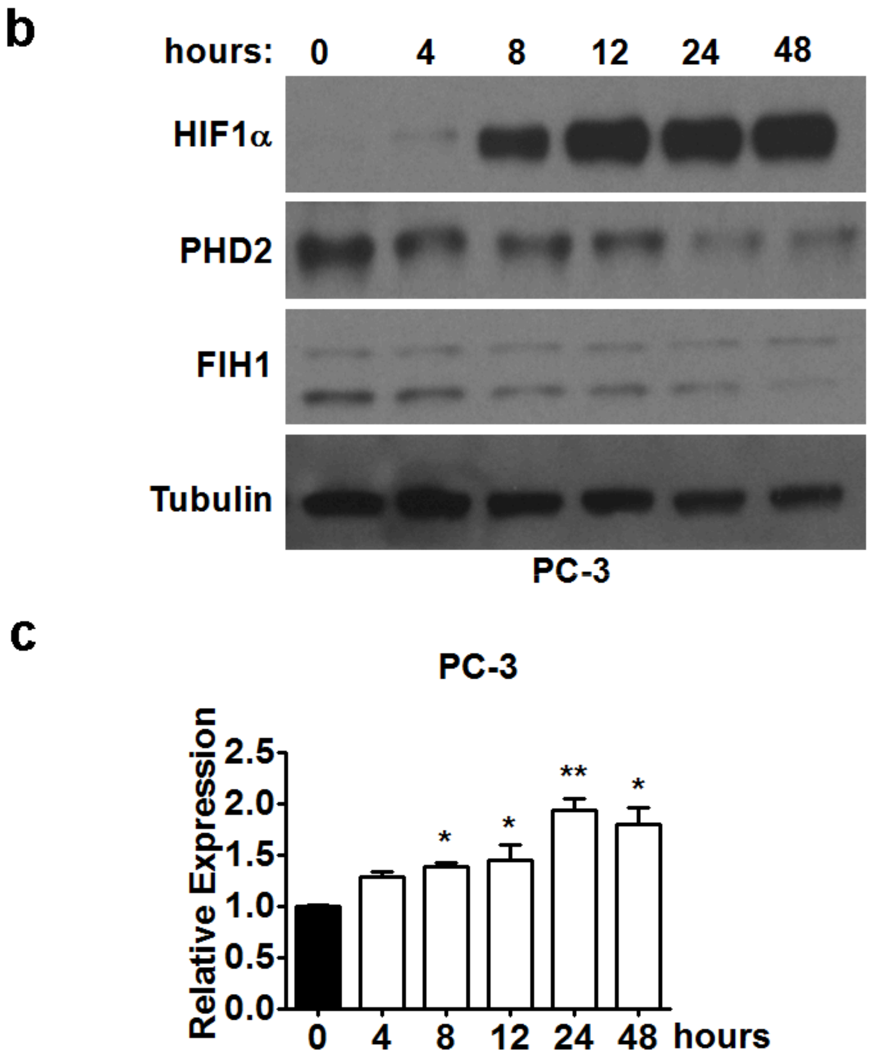

Supplementary Figure. S3.
(a) Mutation (blue character) were introduced into miR-182 regulatory elements in PHD2 and FIH1 3’-UTR. (b, c) Cellular hypoxia was induced in PC-3 cells for different times as indicated. HIF1α, PHD2, and FIH1 protein levels were determined by western blot analysis (b). miR-182 expression was analyzed by RT-PCR (c). Data are mean ± SEM (n=3). *p<0.05, **p<0.01. Full-length blots are presented in Supplementary Fig. S6.

Supplementary Fig. S4

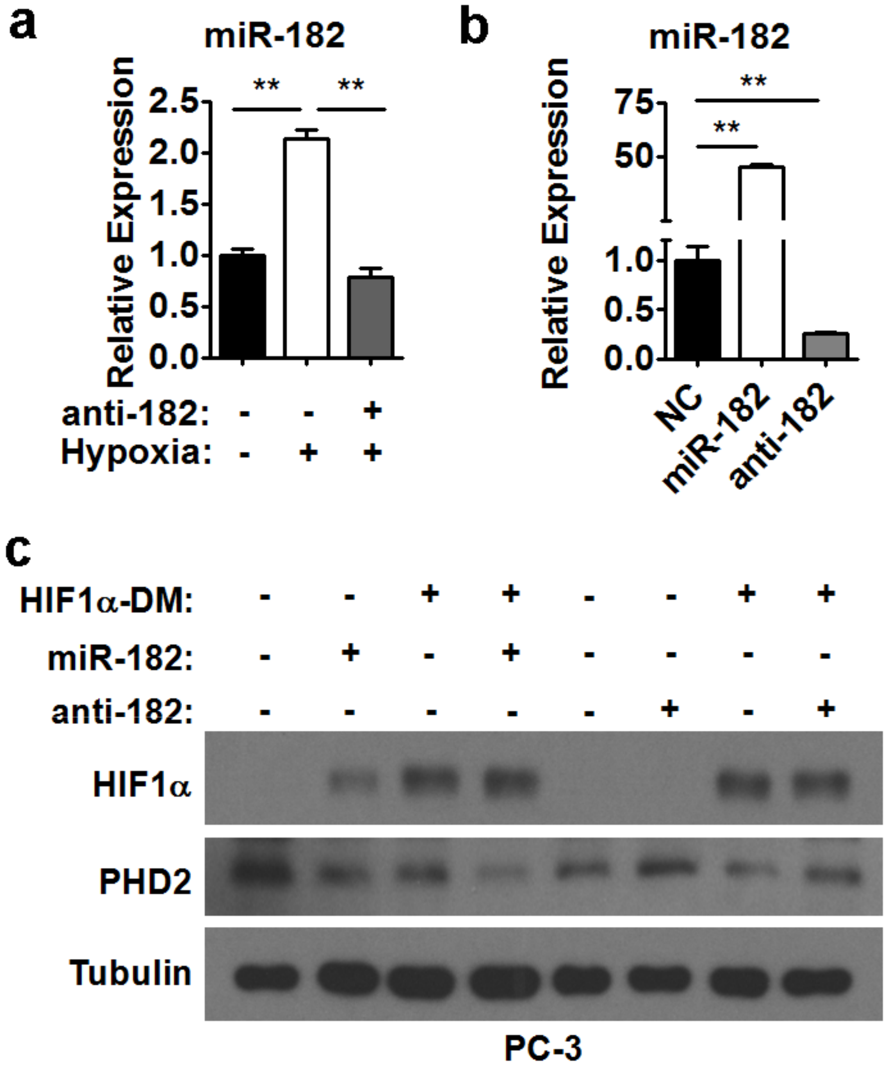

Supplementary Figure. S4.
(a) RT-PCR analysis of miR-182 expression in PC-3 cells transfected with miR-182 antagomir (anti-182) after the induction of cellular hypoxia. (b) RT-PCR analysis of miR-182 expression in PC-3 cells transfected with miR-182 mimics or miR-182 antagomir (anti-182) in a normoxic environment. Data are mean ± SEM (n=3). **p<0.01. (c) PC-3 cells were co-transfected with HIF1α-DM plasmid and miR-182 mimics or miR-182 antagomir (anti-182). 48 hours after transfection, the protein expression of HIF1α and PHD2 were determined by western blot analysis. Full-length blots are presented in Supplementary Fig. S6.

Supplementary Fig. S5

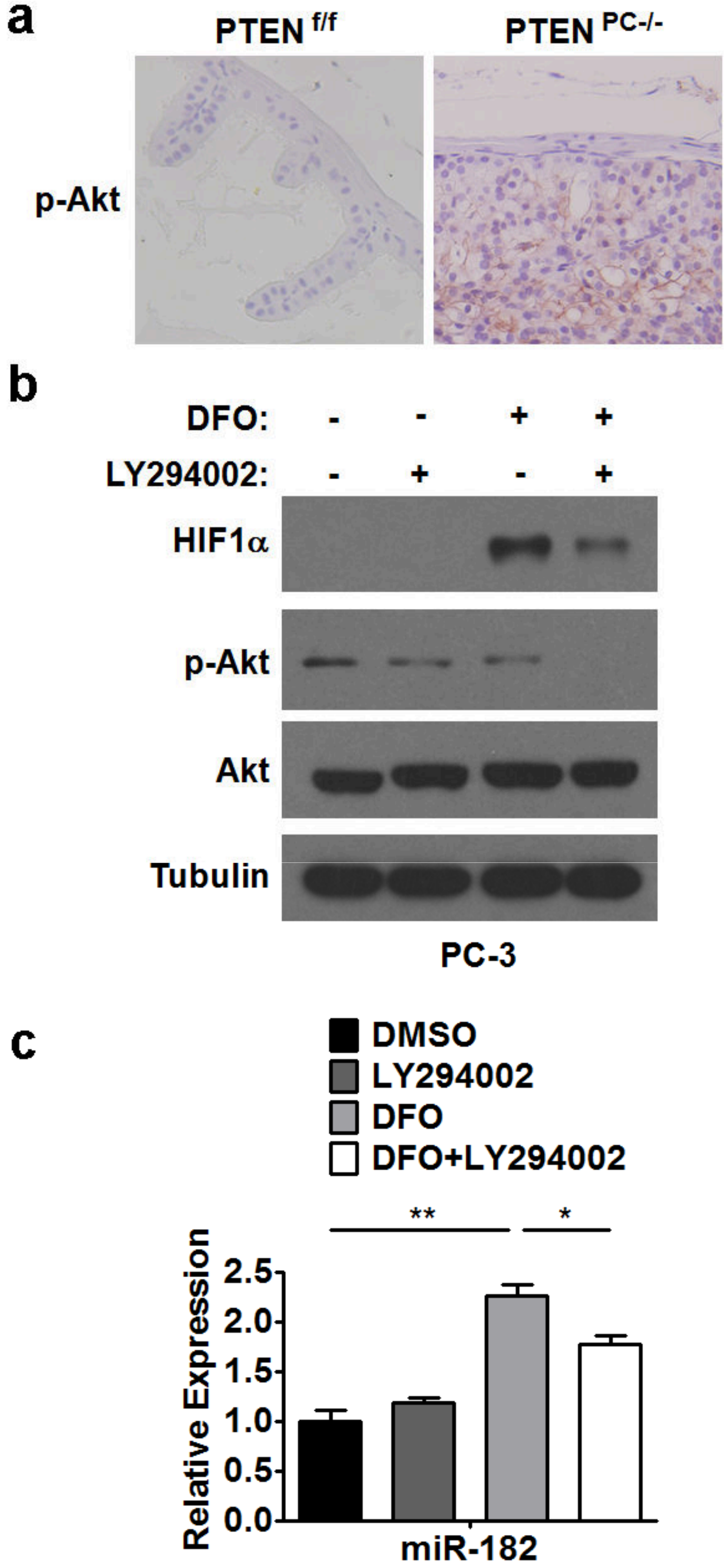

Supplementary Figure. S5.
(a) Immunostaining of p-Akt in the prostate tissues from PTEN $^{f/f}$ and PTEN $^{PC-/-}$ mice. (b, c) Western blot analysis of HIF1α, p-Akt, and total Akt protein expression (b) and RT-PCR analysis of miR-182 expression (c) in PC-3 cells treated with hypoxia inducer DFO and/or PI3K inhibitor LY294002 as indicated for 12 hours. Data are mean ± SEM (n=3). *$p<0.05$, **$p<0.01$. Full-length blots are presented in Supplementary Fig. S6.

Supplementary Fig. S6

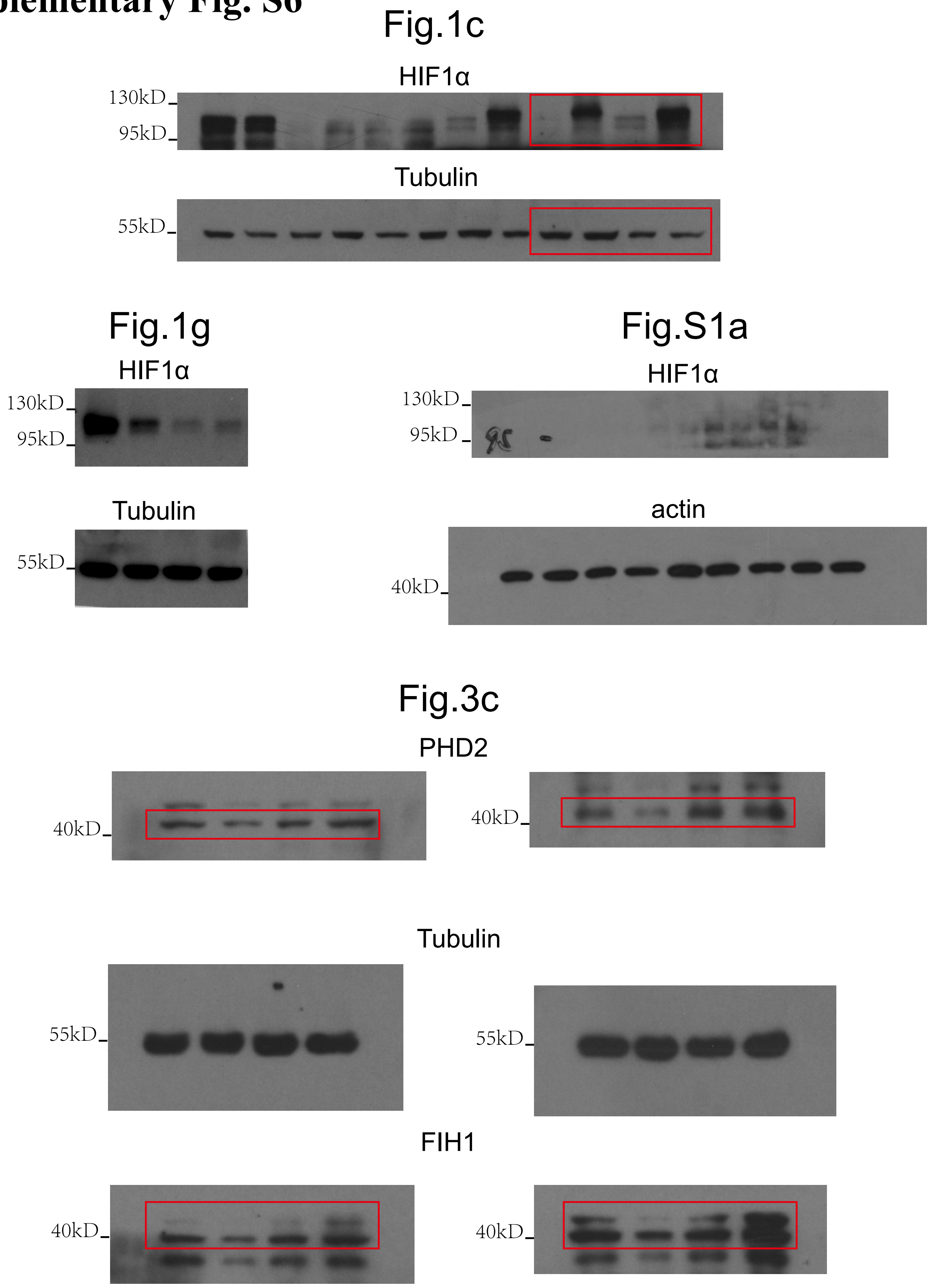

Supplementary Fig. S6

Full-length blots are shown.

Supplementary Fig. S6

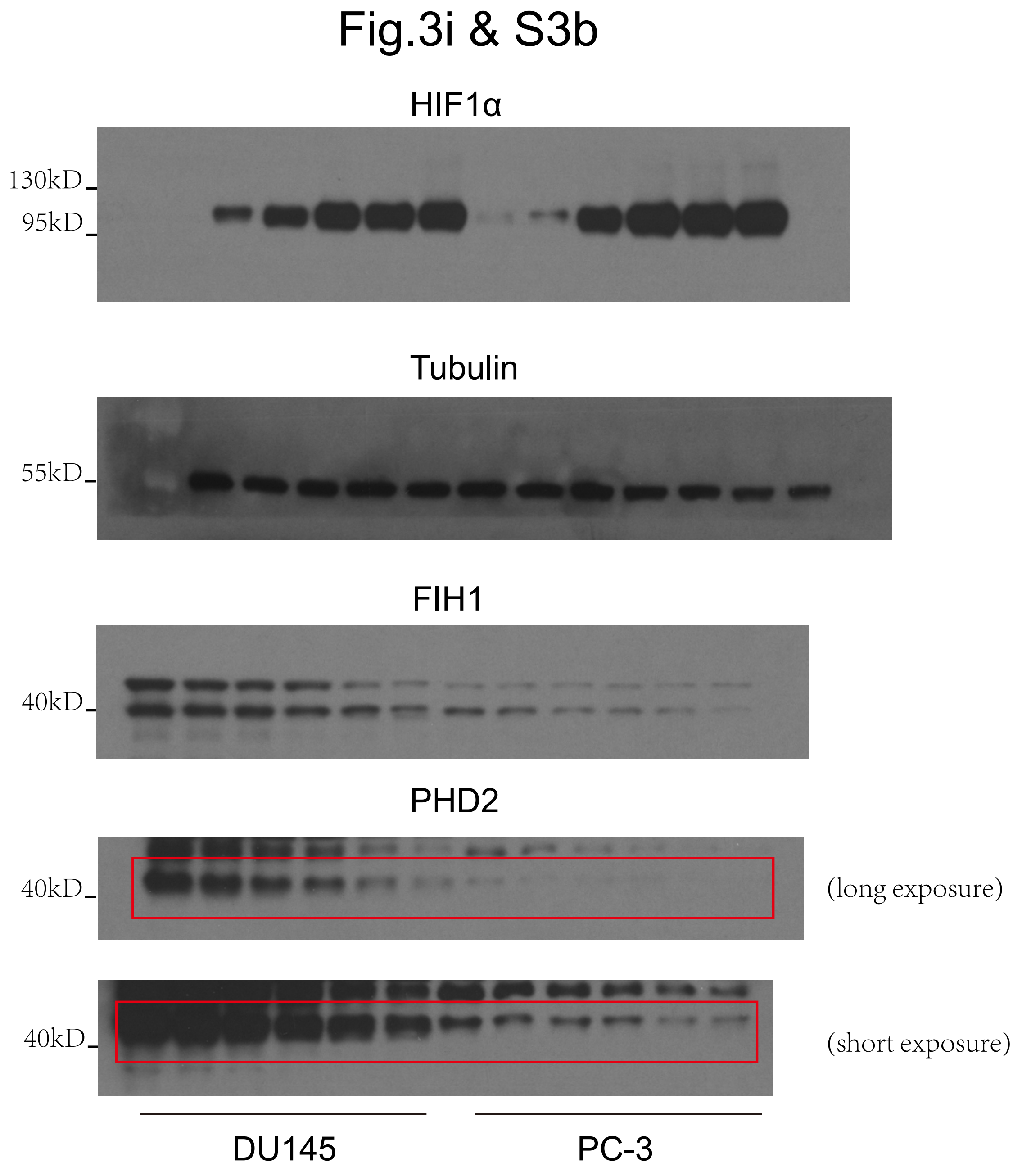

Supplementary Fig. S6

Full-length blots are shown.

Supplementary Fig. S6

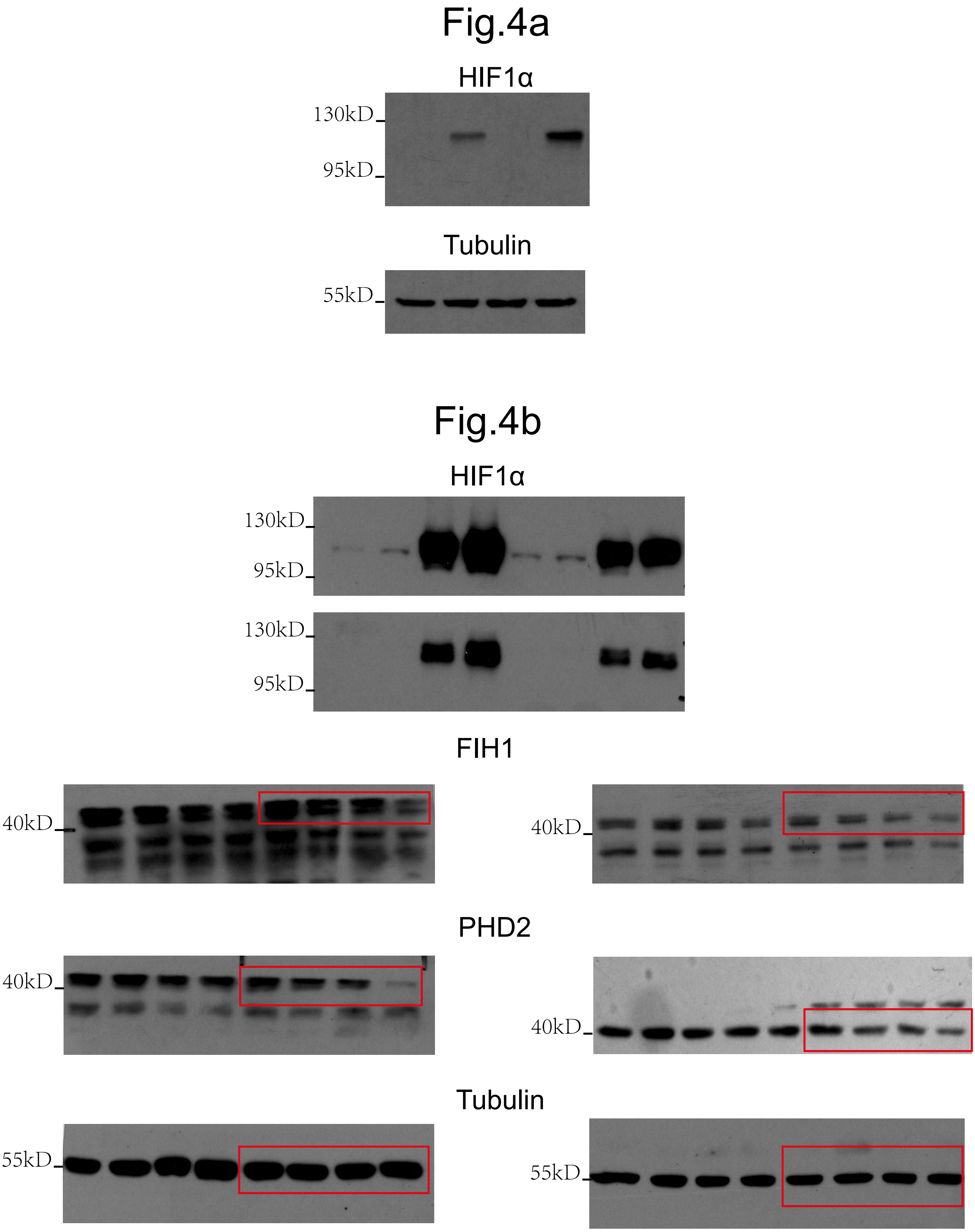

Supplementary Fig. S6

Full-length blots are shown.

Supplementary Fig. S6

Fig.4f

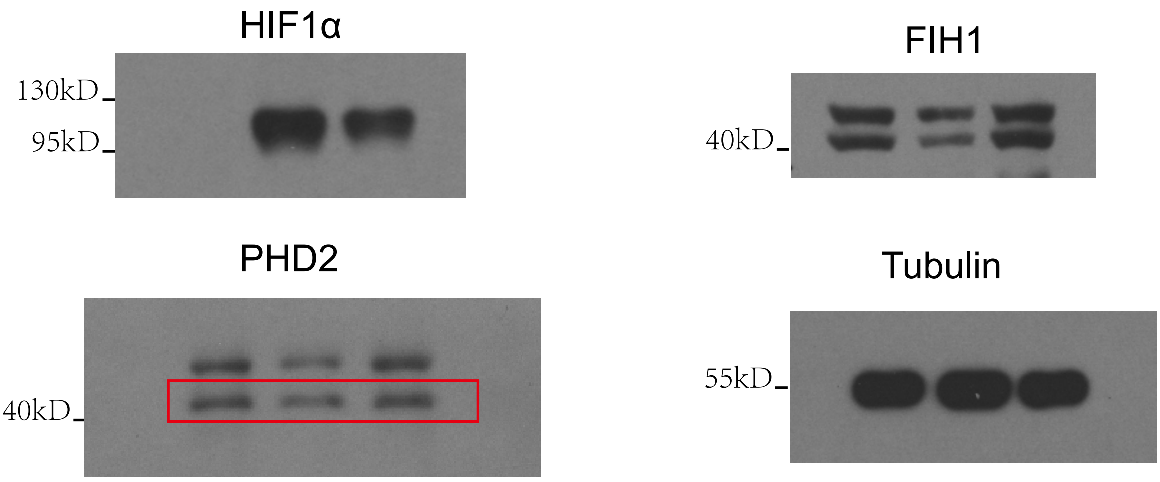

Fig.4g

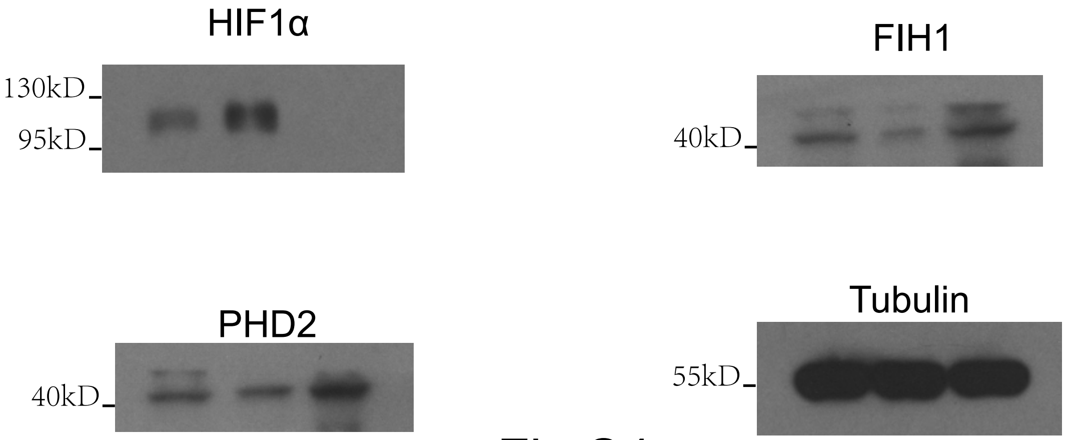

Fig.S4

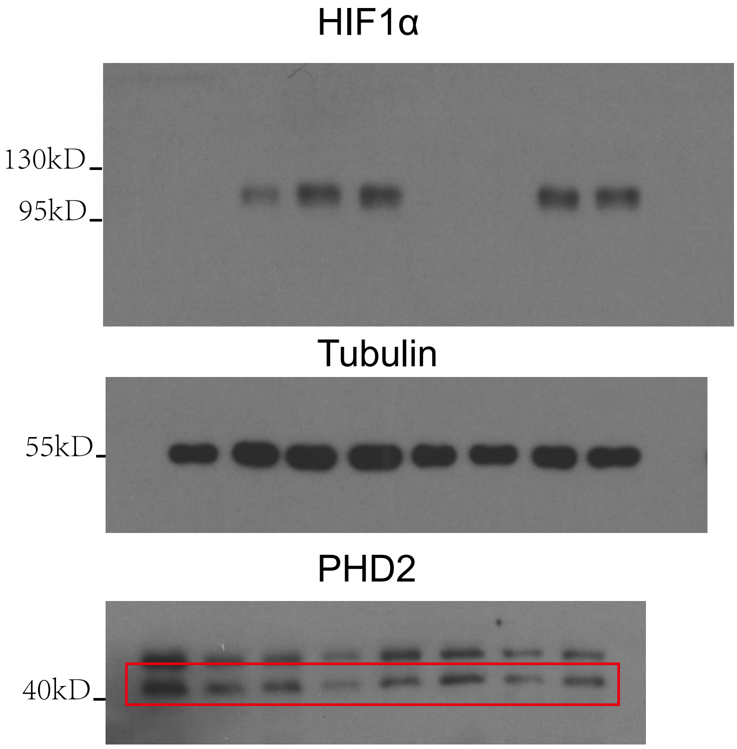

Supplementary Fig. S6

Full-length blots are shown.

Supplementary Fig. S6

Fig.6d

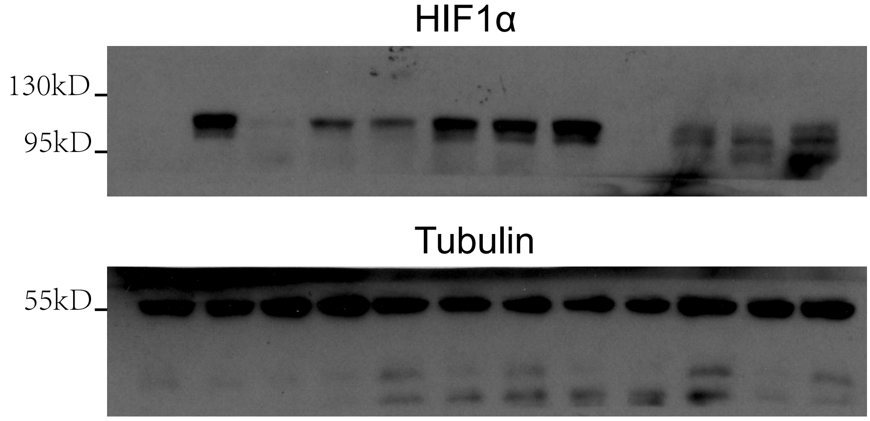

Fig.S5b

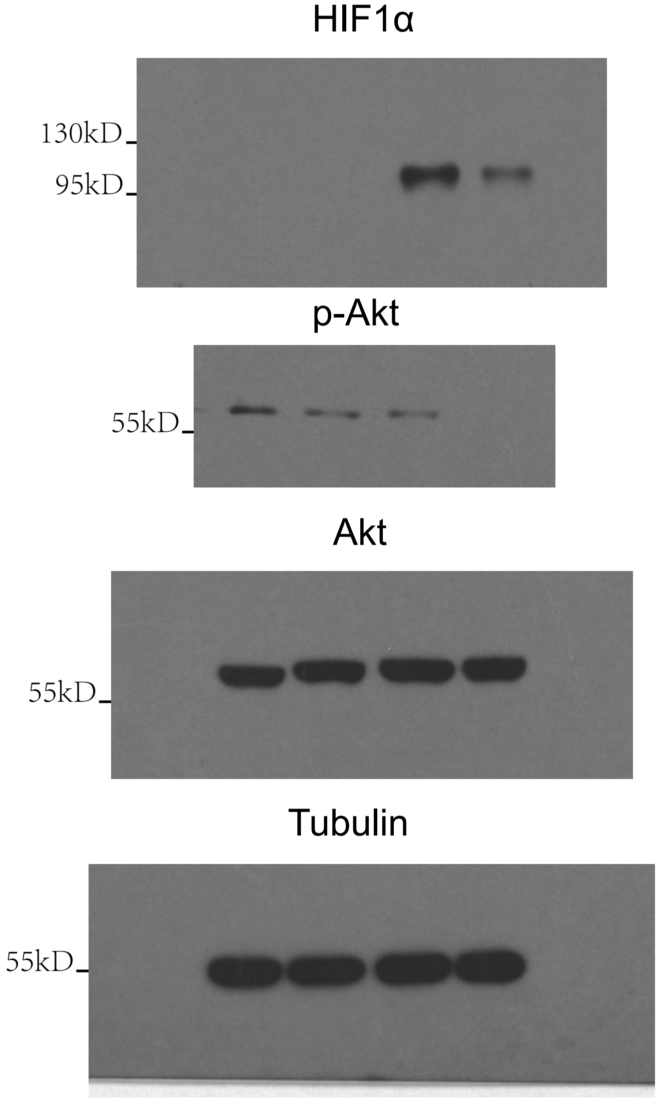

Supplementary Fig. S6

Full-length blots are shown.
